# Definition of a High-Resolution Molecular Marker for Tracking the Genetic Diversity of the Harmful Algal Species *Eucampia zodiacus* Through Comparative Analysis of Mitochondrial Genomes

**DOI:** 10.3389/fmicb.2021.631144

**Published:** 2021-03-24

**Authors:** Mengjia Zhang, Zongmei Cui, Feng Liu, Nansheng Chen

**Affiliations:** ^1^CAS Key Laboratory of Marine Ecology and Environmental Sciences, Institute of Oceanology, Chinese Academy of Sciences, Qingdao, China; ^2^Laboratory of Marine Ecology and Environmental Science, Qingdao National Laboratory for Marine Science and Technology, Qingdao, China; ^3^Institute of Oceanology, University of Chinese Academy of Sciences, Beijing, China; ^4^Center for Ocean Mega-Science, Chinese Academy of Sciences, Qingdao, China; ^5^Department of Molecular Biology and Biochemistry, Simon Fraser University, Burnaby, BC, Canada

**Keywords:** harmful algal bloom species, *Eucampia zodiacus*, mitochondrial genome, genetic marker, comparative genomics

## Abstract

The cosmopolitan phytoplankton species *Eucampia zodiacus* is a common harmful algal bloom (HAB) species that have been found to cause HABs in essentially all coastal regions except the Polar regions. However, molecular information for this HAB species is limited with only a few molecular markers. In this project, we constructed the mitochondrial genome (mtDNA) of *E. zodiacus*, which was also the first mtDNA constructed for any species in the order Hemiaulales that includes 145 reported species (including two additional HAB species *Cerataulina bicornis* and *Cerataulina pelagica*). Comparative analysis of eight *E. zodiacus* strains revealed that they could not be distinguished using common molecular markers, suggesting that common molecular markers do not have adequate resolution for distinguishing *E. zodiacus* strains. However, these *E. zodiacus* strains could be distinguished using whole mtDNAs, suggesting the presence of different genotypes due to evolutionary divergence. Through comparative analysis of the mtDNAs of multiple *E. zodiacus* strains, we identified a new molecular marker *ezmt1* that could adequately distinguish different *E. zodiacus* strains isolated in various coastal regions in China. This molecular marker *ezmt1*, which was ∼400 bp in size, could be applied to identify causative genotypes during *E. zodiacus* HABs through tracking the dynamic changes of genetic diversity of *E. zodiacus* in HABs.

## Introduction

Harmful algal blooms (HABs) are results of rapid algal proliferation and/or aggregation of algae that can cause massive fish deaths, contamination of seafood with toxins, and/or ecological damages through the development of anoxia or habitat alteration (Gentien et al., 2003). HABs have become a global epidemic with significant economic, social, and human health consequences (Gentien et al., 2003). In recent decades, HABs have been increasing their frequency, persistence, regional coverage/spatial extent and economic impact worldwide as a result of enhanced coastal eutrophication, climate change and invasion of alien species (Sarkar, 2018). HAB species are multitudinous but hard to be identified accurately only using traditional morphological examination-based methods (Chen, 2020).

The *Eucampia zodiacus* Ehrenberg is a common HAB species of the genus *Eucampia*, family Hemiaulaceae, order Hemiaulales, class Mediophyceae, and phylum Bacillariophyta. It is 36–72 μm in width and 6–32 μm in height (Jin, 1965; Guo, 2004; Nishikawa and Imai, 2011). Under the light microscope, the alga has an “H” shape in its curved girdle view and it is elliptic in the valve view. The cells are connected by two short, blunt elevations, forming a spiral colony. The plastids are small and numerous, with a small-cake-shape (Hendey, 1964; Yang and Dong, 2006). *E. zodiacus* has a worldwide distribution except for the Polar regions and can be detected almost all-year round in the water column, providing considerable primary production (Horner, 2002; Ito et al., 2013; Nishikawa et al., 2013).

*E. zodiacus* can form dense blooms in coastal waters, which have been observed in the Tokyo Bay (Nishikawa et al., 2011), Harima-Nada (Nishikawa et al., 2007), and Ariake sea (Matsubara, 2012) in Japan, Bay of Fundy (Martin et al., 2008) in Canada, Jiaozhou Bay, Haizhou Bay, Xiangshan Harbour and many other sea areas in China (Huo et al., 2001; Zhang et al., 2002; Liang, 2012). *E. zodiacus* blooms develop and last for a longer time because it is able to grow until the complete exhaustion of the available nutrients in the water column, and can take up as much nitrogen as other species such as *Skeletonema* species at low temperatures (Nishikawa et al., 2009; Ito et al., 2013). Notably, *E. zodiacus* blooms have been reported to cause bleaching of aquacultured nori, fisheries damage and economic losses through algal aggregations, competitive utilizing of nutrients (especially nitrogen) and resultant nutrient depletion in water columns (Martin et al., 2008; Nishikawa et al., 2011).

Notably that *E. zodiacus* blooms displayed both spatial and temporal attributes based on previous studies. For example, *E. zodiacus* blooms often occur in winter and early spring in Japan (Nishikawa et al., 2007), while *E. zodiacus* blooms have been reported to occur most in summer in China (Huo et al., 2001; Zhang et al., 2002; Liang, 2012). Such differential spatial and temporal dynamics of *E. zodiacus* blooms suggest that *E. zodiacus* has genetic diversity and different strains are different in their ability to produce HABs.

Many common molecular markers of *E. zodiacus* including 18S rDNA, 28S rDNA, ITS, *rbcL*, and *CO1* have been sequenced and applied to characterize *E. zodiacus* (Sorhannus, 2007; Rampen et al., 2009; Sorhannus and Fox, 2011; Ashworth et al., 2013; Hamsher et al., 2013; Guo et al., 2015). However, these molecular markers have not been evaluated for their ability to study intra-species genetic diversity of *E. zodiacus*. Some common molecular markers including 18S rDNA, 28S rDNA, and *rbcL* have been used to study intra-species variation (Riisberg and Edvardsen, 2008). However, common molecular markers are usually inadequate for distinguishing intra-species genetic diversity. For example, molecular markers including 18S rDNA, 28S rDNA, ITS, *rbcL*, and *COI* were demonstrated to be ineffective in resolving intra-species genetic diversity in the HAB species *Phaeocystis globosa* (Song et al., 2020). High-resolution molecular markers can be identified through comparative analysis of genomics sequences of the organelle genomes and the nuclear genomes (Song et al., 2020).

Mutation rates differ among mtDNAs, plastid genomes, and nuclear genomes and mutation rates for mtDNAs are usually higher than that for plastid and nuclear genomes. For example, comparative analysis of *Phaeocystis antarctica* and *P. globosa* mtDNAs suggested that the mutation rates for mtDNAs is 10 and 3 times that of the plastid and nucleus, respectively (Smith et al., 2014). Furthermore, mutation rates for intergenic regions are usually much higher than that for genic regions (Guo et al., 2015). As a result, many molecular markers have been developed based on mtDNAs. For example, the molecular marker *MSS* designed for distinguishing different mitotypes in *Brassica napus* help successfully identify 570 different inbred lines collected from various scientific research institutes in China (Heng et al., 2015). However, until now, mtDNAs of only 33 diatoms have been constructed and published, and by now there has been no published mtDNAs in the entire order Hemiaulales, to which *E. zodiacus* belongs. The order Hemiaulales has 145 annotated species including two additional HAB species *Cerataulina bicornis* and *Cerataulina pelagica* according to National Marine Data and Information Service (NMDIS).

We hypothesize that high-resolution molecular markers for analyzing genetic diversity can be developed through comparative analysis of *E. zodiacus* mtDNAs, especially the non-coding sequences that display higher variations. In this study, we constructed the mtDNA of *E. zodiacus* for the first time, demonstrated that common molecular markers including 18S rDNA, 28S rDNA, ITS, *rbcL*, and *CO1* were inadequate for distinguishing *E. zodiacus* strains, and designed a new molecular marker *ezmt1* with high resolution and specificity.

## Materials and Methods

### Strain Isolation, Culturing, and Characterization

**FIGURE 1:**
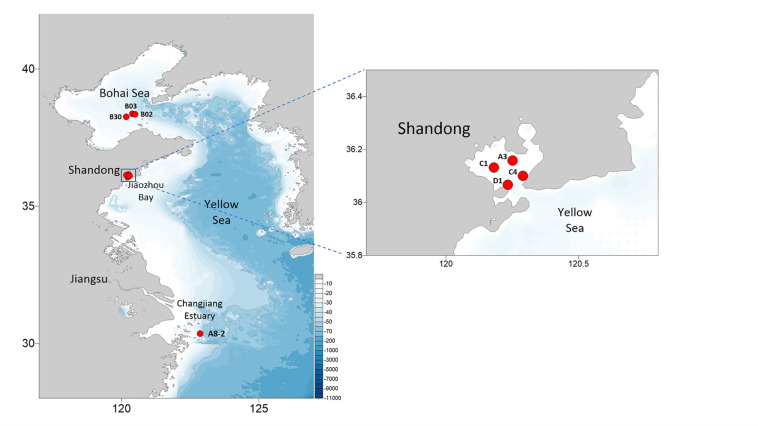
Sampling sites of the eight *E. zodiacus* strains analyzed in this study.

Eight *E. zodiacus* strains (CNS00060, CNS00061, CNS00310, CNS00311, CNS00312, CNS00313, CNS00314, and CNS00315) were individually isolated from seawater samples collected during expeditions in multiple coastal regions in China, including the Jiaozhou Bay (August, 2019 and January, 2020) on the research vehicle “Chuangxin” operated by the Jiaozhou Bay Marine Ecosystem Research Station, the Changjiang Estuary (July, 2019) on the research vehicle “Zheyu 2” supported by the Natural Science Foundation of China (NSFC), and the Bohai Sea (October, 2019) on the research vehicle “Beidou” supported by the National Natural Science Foundation of China, Bohai and Yellow Sea Oceanography Expedition (NORC2019-01) (Figure 1). Briefly, phytoplankton cells were individually selected with a micropipette, followed by repeated washes before being transferred to 24-well culture dishes. They were then transferred to cell culture flask (60–750 ml) to accumulate enough biomass for further molecular assays. Phytoplankton cells were grown in L1 seawater culture medium and maintained with temperature of 18–20°C, irradiance of 30 μM photons m^–2^ s^–1^ and photoperiod of 12/12-h light/dark.

For morphological identification, cells were mounted on the glass-slide and observed with a ZEISS IMAGER A2 microscope equipped with differential interference contrast optics (Hadziavdic et al., 2014). For molecular identification, sequences of five common molecular markers, including full-length 18S rDNA, 28S rDNA D1-D2, ITS, *COI*, and *rbcL* were sequenced using Sanger sequencing technology after PCR amplification using primers listed in Table 1. PCR conditions for amplifying 18S rDNA began with a denaturation at 94°C for 4 min, followed by 32 cycles of (denaturation at 94°C for 1 min, annealing at 57°C for 1:50, extension at 72°C for 2 min), and a final extension at 72°C for 10 min (Saldarriaga et al., 2003). PCR conditions for amplifying 28S rDNA D1–D2 began with a denaturation at 94°C for 5 min, followed by 35 cycles of (denaturation at 94°C for 30 s, annealing at 60°C for 30 s, extension at 72°C for 50 s), and a final extension at 72°C for 10 min (Lundholm et al., 2002). PCR conditions for amplifying ITS began with a denaturation at 94°C for 5 min, followed by 35 cycles of (denaturation at 94°C for 40 s, annealing at 58°C for 40 s, extension at 72°C for 1 min), and a final extension at 72°C for 10 min (Utama et al., 2017). PCR conditions for amplifying *rbcL* began with a denaturation at 94°C for 5 min, followed by 35 cycles of (denaturation at 94°C for 50 s, annealing at 53°C for 50 s, extension at 72°C for 1:10), and a final extension at 72°C for 10 min (Alverson et al., 2007). PCR conditions for amplifying *COI* began with a denaturation at 94°C for 5 min, followed by 35 cycles of (denaturation at 94°C for 30 s, annealing at 50°C for 1 min, extension at 72°C for 1:10), and a final extension at 72°C for 10 min.

**TABLE 1:** Oligonucleotide primers used to amplify and sequence 18S rDNA, 28S rDNA, ITS, *COI*, and *rbcL* fragments from *E. zodiacus.*

Name	Marker	Sequence (5′–3′)	References
28F	*SSU*	CGA ATT CAA CCT GGT TGA TCC TGC CAG T	Saldarriaga et al., 2003
42R	*SSU*	CCG GAT CCT GAT CCT TCT GCA GGT TCA CCT AC	Saldarriaga et al., 2003
R-582	*SSU*	AAT TAC CGC GGC TGC TGG CAC CV	Hadziavdic et al., 2014
F-898	*SSU*	AGA GGT GAA ATT CTY RGA	Hadziavdic et al., 2014
R-1200	*SSU*	CCC GTG TTG AGT CAA ATT AAG C	Hadziavdic et al., 2014
F-1422	*SSU*	ATA ACA GGT CTG TGA TGC CC	Hadziavdic et al., 2014
D1R-F	*LSU* d1-d2	ACC CGC TGA ATT TAA GCA TA	Lundholm et al., 2002
D2C-R	*LSU* d1-d2	CCT TGG TCC GTG TTT CAA GA	Lundholm et al., 2002
ITS1	*ITS*	TCC GTA GGT GAA CCT GCG G	Utama et al., 2017
ITS4	*ITS*	TCC TCC GCT TAT TGA TAT GC	Utama et al., 2017
rbcL66+	*rbcL*	TTA AGG AGA AAT AAA TGT CTC AAT CTG	Alverson et al., 2007
rbcL1255−	*rbcL*	TTG GTG CAT TTG ACC ACA GT	Alverson et al., 2007
rbcL527+	*rbcL*	AAA ACA TTC CAA GGT CCT GCT	Alverson et al., 2007
rbcL587−	*rbcL*	GTC TAA ACC ACC TTT TAA MCC TTC V	Alverson et al., 2007
Z3COI-F	*COI*	GGC AAC AGG AAC TAA TCT T	This study
Z3COI-R	*COI*	CTA CTA GAA GAC AAT GCT TC	This study

*+ Forward PCR amplification primer; − Reverse PCR amplification primer.*

### DNA Library Preparation and Whole Genome Sequencing

Cultures at the exponential growth phase were harvested and concentrated via centrifugation, followed by total nucleic acids extraction with TIANGEN DNAsecure Plant Kit (TIANGEN, DP121221). Genomic DNA sample was fragmented by sonication via set program to a size of 350 bp. Then a single adenosine “A” was added to the 3′ end of the double-stranded DNA after end modification to prevent the self-connection of the flat ends between DNA fragments, and it can also highlight the complementary pairing with the single “T” at the 5′ end of the next sequencing connector for accurate connection, effectively reducing the self-connection between library fragments. DNA fragments were then ligated with the full-length adapter for Illumina sequencing, followed by further PCR amplification. After PCR products were purified by AMPure XP system (Beckman Coulter, Beverly, United States), DNA concentration was measured by Qubit^®^3.0 Flurometer (Invitrogen, United States), libraries were analyzed for size distribution by NGS3K/Caliper and quantified by real-time PCR (3 nM). After cluster generation, the DNA libraries were sequenced on Illumina Novaseq 6000 platform and 150 bp paired-end reads were generated. The whole genome sequencing was finished at Novogene (Beijing, China).

### Construction of mtDNA

Raw data were filtered into clean data with FASTQ following the rules (1) identifying and removing reads with tail pollution; (2) removing reads with low quality (>50% bases having Phred quality < 5) and (3) removing reads with ≥ 10% unidentified nucleotides (N). The filtered reads were assembled into scaffolds with Platanus-allee (v2.2.2) (Kajitani et al., 2019) with default parameters, ABySS (v2.2.4) (Jackman et al., 2017) with the option *k* = 96 and SPAdes (v3.14.0) (Bankevich et al., 2012) with default parameters. With the mtDNA of *Skeletonema marinoi* (NC_028615) (An et al., 2017) and *Thalassiosira pseudonana* (NC_007405) (Armbrust et al., 2004) serving as references, scaffolds corresponding to mtDNA of *E. zodiacus* were identified using BLAST with the option e-value = 0.00001, max_target_seqs = 100. When achieving one scaffold only, we then used MEGA (v7.0) (Matus et al., 2014) and DOTTER (v4.44.1) to estimate whether sequences at the ends achieved overlap. Draft mtDNA sequence was constructed by merging the ends by taking advantage of the overlapping segments at the ends. If no overlapping sequences were identified, draft mtDNA sequence was formed by substituting gaps with a stretch of N. Reads were then aligned to the draft mtDNA sequence using BWA (v0.7.17-r1188) (Li and Durbin, 2009) with default parameters, results of which were extracted with SAMtools (v1.10) (Li et al., 2009) and viewed with IGV (v2.7.2) (Robinson et al., 2011). According to alignments, assembly errors were corrected and N regions were replaced. The final version of the mtDNA was validated through an additional round of alignment with BWA and visualization with IGV. Of all filtered clean sequence data, 1.24% represented mtDNA, while contamination accounted for 0.33%.

### mtDNA Annotation

Protein-coding genes (PCGs) and open reading frames (*orfs*) were annotated using NCBI ORF Finder and BLAST similarity searches of the non-redundant databases at NCBI (Altschul et al., 1997). tRNAs were determined by reconstructing their cloverleaf structures using the tRNAscan-SE (v1.3.1) (Lowe and Chan, 2016) with default parameters. rRNAs were identified using RNAmmer (v1.2) (Lagesen et al., 2007), Barrnap (v0.9) and MEGA (v7.0) for homologous comparison. The gene map of the circular mtDNA of *E. zodiacus* was generated with Organellar Genome DRAW (OGDraw) (Lohse et al., 2007). The mtDNA sequence of *E. zodiacus* strain CNS00060 has been deposited in GenBank with the accession number of MW026607.

For accurate comparative analysis of genes of mtDNAs of 33 diatom species in Bacillariophyta, we re-annotated all of these 33 published mtDNAs (Table 2) by searching for missing genes and correcting annotation errors. Nucleotide composition was calculated using DNA Sequence Polymorphism (DnaSP) software (v6.0) (Rozas et al., 2017).

**TABLE 2:** Genome information of 34 mitogenomes from the Phylum Bacillariophyta for comparative analysis.

Class	Species	Strain	Habitat	Accession number	Size (bp)	A + T (%)	References
Mediophyceae (4)	*Eucampia zodiacus*	CNS00060	Marine	MW026607	36,162	74.9	This study
	*Skeletonema marinoi*	voucher 06.JK029	Marine	NC_028615	38,515	70.3	An et al., 2017
	*Thalassiosira pseudonana*	–	Marine	NC_007405	43,827	69.9	Armbrust et al., 2004
	*Toxarium undulatum*	ECT3802	Marine	NC_037988	40,429	69.9	Guillory et al., 2018
Coscinodiscophyceae (1)	*Melosira undulata*	–	Freshwater	NC_037728	32,777	78.4	Pogoda et al., 2019
Bacillariophyceae (29)	*Asterionella formosa*	BGM1	Freshwater	NC_032029	61,877	73.3	Villain et al., 2017
	*Synedra acus*	–	Freshwater	NC_013710	46,657	68.3	Ravin et al., 2010
	*Psammoneis japonica*	–	Marine	NC_037989	73,622	69.2	Guillory et al., 2018
	*Cylindrotheca closterium*	CCMP1855	Marine	NC_037986	37,784	67.9	Guillory et al., 2018
	*Fragilariopsis kerguelensis*	–	Marine	LR812619	37,348	68.6	–
	*Nitzschia palea*	–	Freshwater	MH297491	37,754	69.1	Crowell et al., 2019
	*Nitzschia palea* (nearly complete)	NIES-2729	Freshwater	AP018512	>36,830	–	Kamikawa et al., 2018
	*Nitzschia alba*	–	Marine	NC_037729	36,252	71.6	Pogoda et al., 2019
	*Nitzschia* sp.	PL1-4	–	AP018507	38,056	69.5	Kamikawa et al., 2018
	*Nitzschia* sp.	NIES-3576	–	AP018509	37,792	69.8	Kamikawa et al., 2018
	*Nitzschia* sp.	4	–	NC_037990	36,012	71.1	Guillory et al., 2018
	*Nitzschia* sp.	NIES-3581	–	AP018510	35,897	70.8	Kamikawa et al., 2018
	*Nitzschia* sp. (nearly complete)	PL3-2	–	AP018505	>35,839	–	Kamikawa et al., 2018
	*Pseudo-nitzschia multiseries*	–	Marine	NC_027265	46,283	68.9	Yuan et al., 2016
	*Didymosphenia geminata*	–	Freshwater	NC_032171	37,765	73.1	Aunins et al., 2018
	*Entomoneis* sp.	–	–	MF997419	36,078	72.2	Pogoda et al., 2019
	*Halamphora calidilacuna*	–	Marine	MF997424	103,605	68.8	Pogoda et al., 2019
	*Halamphora coffeaeformis*	–	Brackish	NC_037727	44,653	67.1	Pogoda et al., 2019
	*Berkeleya fennica*	–	Freshwater	NC_026126	35,509	70.2	An et al., 2016a
	*Fistulifera solaris*	–	Marine	NC_027978	39,476	71.9	Tang and Bi, 2016
	*Haslea nusantara*	–	Marine	NC_044492	36,288	70.8	Prasetiya et al., 2019
	*Navicula ramosissima*	voucher 10.TA439	Marine	NC_031848	48,652	68.9	An et al., 2016b
	*Phaeodactylum tricornutum*	ICE-H	Marine	MN956530	77,055	65.3	–
	*Phaeodactylum tricornutum*	–	Marine	NC_016739	77,356	65.0	Secq and Green, 2011
	*Proschkinia* sp.	SZCZR1824	–	MH800316	48,863	70.4	Gastineau et al., 2019
	*Surirella* sp.	–	–	MF997423	42,867	72.6	Pogoda et al., 2019
	Endosymbiont of *Kryptoperidinium foliaceum* (partial)	–	–	JN378734	>39,686	–	Imanian et al., 2012
	Endosymbiont of *Durinskia baltica* (partial)	–	–	JN378735	>35,505	–	Imanian et al., 2012
	*Eunotia naegelii*	UTEX FD354	Freshwater	NC_037987	48,049	72.9	Guillory et al., 2018

The PCGs were extracted from the mtDNAs using BedTools (v2.28.0) (Quinlan and Hall, 2010), the same of which from all 34 diatoms was aligned using MAFFT (v7.471-1) (Katoh and Standley, 2013) with default parameters. The ambiguously aligned regions in each alignment were removed using trimAl (v1.4) (Capella-Gutierrez et al., 2009) with the option gt = 1, and all genes from each diatom were then concatenated with the same order using Phyutility (v2.7.1) (Smith and Dunn, 2008). The set of 32 PCGs shared among the 34 Bacillariophyta mtDNAs were used for phylogenetic analysis, including *atp6*, *8*, *9*; *cob*; *cox1*, *2*, *3*; *nad1-7*, *4L*, *9*, *11*; *rpl2*, *5*, *6*, *14*, *16*; *rps3*, *4*, *8*, *10*, *11*, *13*, *14*, *19*; and *tatA*, *tatC*. Phylogenetic relationships were evaluated based on the amino acid (aa) sequence dataset of these 32 PCGs. Mitochondrial genes of two species *Phytophthora ramorum* (DQ832718) and *Saprolegnia ferax* (AY534144) in Oomycota were selected as out-groups (Liu et al., 2019). The evolutionary relationship was inferred by using the maximum likelihood (ML) method, conducted by IQ-TREE (v1.6.12) (Trifinopoulos et al., 2016) with 1,000 bootstrap replicates. The best-fit models for each partition were determined automatically using IQ-TREE with the subroutine ModelFinder. According to the tree, mtDNAs of *S. marinoi*, *T. pseudonana*, and *E. zodiacus* were selected for multiple sequence alignment using Mauve Genome Alignment (v2.3.1) (Darling et al., 2010) with progressive Mauve algorithm. Pairwise comparison of the three was shown in the CIRCOS (v0.69) (Krzywinski et al., 2009).

### Single Nucleotide Variants (SNVs) Detection in mtDNAs of *E. zodiacus* Strains

Phylogenetic tree based on the whole mtDNAs showed genomic diversity. To search for genomic variations (GVs), we aligned Illumina sequencing clean reads of the seven *E. zodiacus* strains against the mtDNA of the reference strain CNS00315 using BWA with default parameters. Alignment results were screened using SAMtools with default parameters, and SNVs with homozygous support > 85% were called using VarScan (v2.4.4) (Koboldt et al., 2012) with the option –min-freq-for-hom = 0.85.

### Searching for High-Density SNVs Regions for Designing High-Resolution Molecular Markers

SNV positions of seven strains were integrated relative to mtDNA of strain CNS00315 using in-house Python scripts, which were also developed to scan for variations in 400 bp-sliding (the length was appropriate for metabarcoding projects using Illumina DNA sequencing technology) successive windows (spaced at 1 bp) along the mtDNA of CNS00315. Each window was evaluated for SNV density and its ability to resolve eight *E. zodiacus* strains. The results were displayed using CIRCOS. To amplify the identified molecular marker, we use the forward primer: MCCCTATGGTATTAGAGA, and the reverse primer: RTTAAGTGACCCAAGTTCTAAG. PCR amplifications in reaction mixtures (final volume, 50 μl) began with a 5 min denaturation step at 94°C, which was followed by 35 cycles of denaturation at 94°C for 30 s, annealing at 45°C for 1 min, and extension at 72°C for 1 min and then by a final extension at 72°C for 10 min.

## Results

### Morphological and Molecular Identification of *E. zodiacus* Strains

**FIGURE 2:**
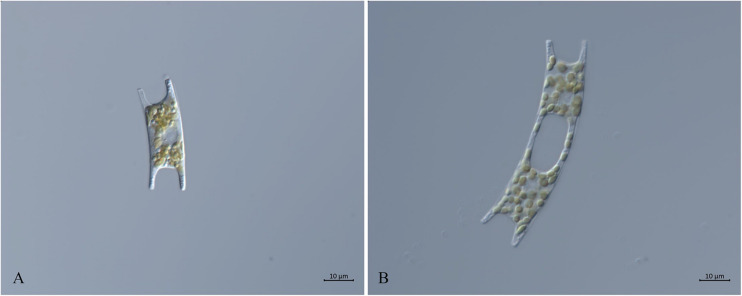
Micrographs of *E. zodiacus* Strain CNS00060 (broad girdle view, live material DIC). **(A)** Single cell with small and numerous plastids. **(B)** Two connected single cells.

Eight *E. zodiacus* strains collected in the Jiaozhou Bay, the Changjiang Estuary and the Bohai Sea were first identified based on their morphological features observed using light microscopy. These cells were generally “H” shaped with small and numerous plastids, with the middle part of each girdle being concave (Figure 2A). Both ends of the apical axis had elevations, with single-cells connected and forming a spiral population with small intercellular space (Figure 2B). The morphological features were consistent with published observations of *E. zodiacus* (Guo, 2004; Yang and Dong, 2006).

The strains were further examined and compared molecularly using five common molecular markers including full-length 18S rDNA, 28S rDNA D1-D2 region, ITS, *COI*, and *rbcL*. All eight strains shared the same percent identity (PID, which was used to quantify the similarity between the biomolecular sequence) (99.94%) when compared to the reference sequence of *E. zodiacus* on full-length 18S rDNA (Sorhannus, 2007). Similar high PIDs were found for other molecular markers including 28S rDNA D1-D2 (100%) (Hamsher et al., 2013), ITS (99.28%) (Guo et al., 2015), *CO1* (99.25%) (Guo et al., 2015), and *rbcL* (100%) (Guo et al., 2015), respectively. Phylogenetic analysis of molecular marker sequences obtained for all eight strains indicated that they all clustered well with corresponding *E. zodiacus* sequence records at GenBank (Supplementary Figure S1), further confirming that these strains were all indeed *E. zodiacus*. However, none of these common molecular markers could distinguish these 8 *E. zodiacus* strains, suggesting that their resolution was limited in distinguishing intra-species genetic diversity.

### General Characteristics of the *E. zodiacus* mtDNA

**FIGURE 3:**
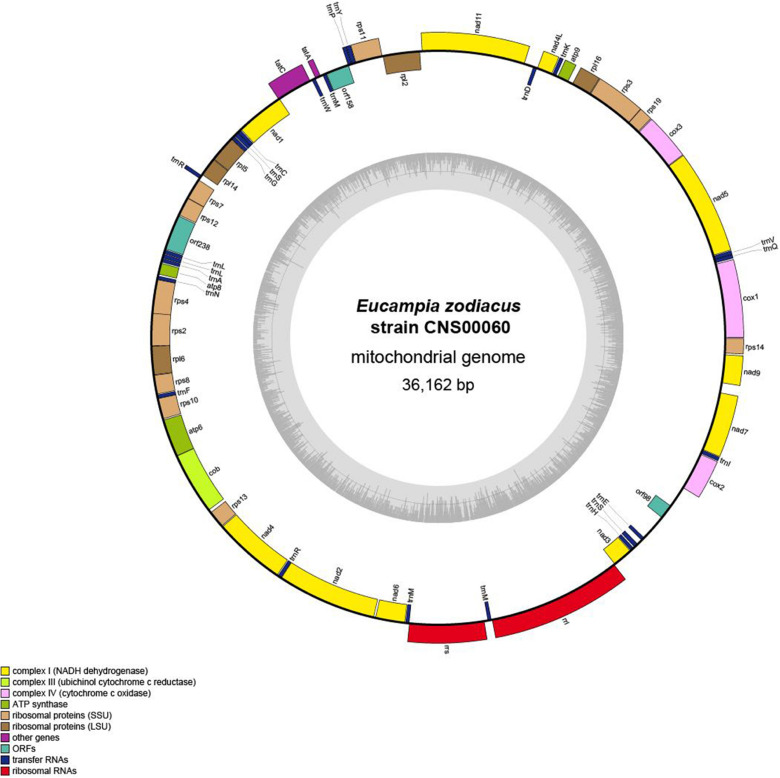
Gene map of the *E. zodiacus* Strain CNS00060. Genes shown on the inside of the map are transcribed in a clockwise direction, whereas those on the outside of the map are transcribed counterclockwise. The assignment of genes into different functional groups is indicated by different colors. The ring of bar graphs on the inner circle shows the GC content in dark gray.

The complete mtDNA of *E. zodiacus* (strain CNS00060) was a circular molecule that was 36,162 bp in size (Figure 3), which was similar to but smaller than the mtDNAs of most diatoms (Table 2). The compact genome size of *E. zodiacus* was primarily due to small intergenic regions (Ravi et al., 2018). Total intergenic regions in *E. zodiacus* mtDNA had a total size of 2,495 bp (only accounting for 6.9%). Three pairs of genes overlapped with each other, including *rps4-rps2* (20 bp), *nad1-tatC* (20 bp), and *orf158-trnP* (9 bp). The first two were synthetic and the last one was reversed. Additionally, three pairs of genes were connected directly without space, including *rps19-rps3*, *rps7-rps12*, and *atp6-cob*. No introns were identified in the *E. zodiacus* mtDNA. We found a pair of small inverted repeat (IR) region (129 bp) on either side of the *orf98* in the intergenic regions.

The AT content of the *E. zodiacus* mtDNA was 74.9%, which was higher than that of most diatom mtDNAs (Table 2). The distribution of genes on the two strands was uneven, with the number of genes on one strand about 1.5 times of those on the other strand. Although the diatom mtDNA sizes varied substantially with different number of nucleotides in non-coding sequences, the recorded diatom mtDNAs had a highly similar gene content. All PCGs commenced with a methionine start codon, expect for the gene *atp8*, which started with ATC. Start codons of *atp8* also varied in many other diatoms (Table 3). The *E. zodiacus* mtDNA is relatively compact, compassing 35 PCGs, 24 tRNAs, 2 rRNAs, and 3 *orfs* of unknown functions. All of the sequenced diatom mtDNAs shared 34 core genes, including 32 PCGs (*atp6*, *atp8*, *atp9*, *cob*, *cox1*, *cox2*, *cox3*, *nad1*, *nad2*, *nad3*, *nad4*, *nad5*, *nad6*, *nad7*, *nad4L*, *nad9*, *nad11*, *rpl2*, *rpl5*, *rpl6*, *rpl14*, *rpl16*, *rps3*, *rps4*, *rps8*, *rps10*, *rps11*, *rps13*, *rps14*, *rps19*, *tatA*, and *tatC*) and two rRNAs (*rnl* and *rns*) (Table 3). In addition to these core genes, we also found *rps2* (which was lost in *Synedra acus* mtDNA and *Melosira undulata* mtDNA), *rps12* (which was lost in *Halamphora calidilacuna* mtDNA) and *rps7* (which was lost in *Pseudo-nitzschia multiseries* mtDNA) in the mtDNA of *E. zodiacus*. The gene *rrn5*, which was found in many diatom genomes, was absent from the mtDNA of *E. zodiacus*. While the gene *nad11* is split into two parts most in many species in Bacillariophyceae, the *nad11* gene in the *E. zodiacus* mtDNA harbored a full *nad11* protein, similar to species in Mediophyceae and Coscinodiscophyceae.

**TABLE 3:** Mitochondrial gene content in 34 mitogenomes from Bacillariophyta.

Species	34 core genes		*rps2*	*rps7*	*rps12*	*rrn5*	tRNA	Introns (I/II)	*nad11* split coding region	Start codon of *atp8*
	32 PCGs	2 rRNAs								
*Eucampia zodiacus*	+	+	+	+	+	−	24	0	−	ATC
*Skeletonema marinoi* voucher 06.JK029	+	+	+	+	+	−	25	0	−	GTG
*Thalassiosira pseudonana*	+	+	+	+	+	−	25	0/1	−	ATT
*Toxarium undulatum* strain ECT3802	+	+	+	+	+	−	26	0	−	ATG
*Melosira undulata*	+	+	−	+	+	−	24	0	−	ATT
*Asterionella formosa* strain BGM1	+	+	+	+	+	−	24	0/1	−	TTG
*Synedra acus* (*Ulnaria acus*)	+	+	−	+	+	−	24	0/3	−	ATG
*Psammoneis japonica*	+	+	+	+	+	−	28	0/11	−	ATG
*Cylindrotheca closterium* strain CCMP1855	+	+	+	+	+	−	24	0/1	+	ATA
*Fragilariopsis kerguelensis*	+	+	+	+	+	−	24	0	+	ATA
*Nitzschia palea*	+	+	+	+	+	−	24	0	+	ATG
*Nitzschia palea* NIES-2729 (nearly complete)	+	+	+	+	+	−	24	0	+	ATG
*Nitzschia alba*	+	+	+	+	+	−	24	0	+	ATG
*Nitzschia* sp. PL1-4	+	+	+	+	+	−	24	0	+	ATG
*Nitzschia* sp. NIES-3576	+	+	+	+	+	−	24	0	+	ATG
*Nitzschia* sp. strain 4	+	+	+	+	+	−	24	0	+	ATG
*Nitzschia* sp. NIES-3581	+	+	+	+	+	−	24	0	+	ATG
*Nitzschia* sp. PL3-2 (nearly complete)	+	+	+	+	+	−	24	0	+	ATG
*Pseudo-nitzschia multiseries*	+	+	+	−	+	−	24	0/3	+	ATA
*Didymosphenia geminata*	+	+	+	+	+	−	25	0	+	ATG
*Entomoneis* sp.	+	+	+	+	+	−	23	0	+	ATG
*Halamphora calidilacuna*	+	+	+	+	−	+	26	1/19	+	ATG
*Halamphora coffeaeformis*	+	+	+	+	+	+	24	0/5	+	ATG
*Berkeleya fennica*	+	+	+	+	+	+	25	0	+	ATG
*Fistulifera solaris*	+	+	+	+	+	+	24	0	+	ATG
*Haslea nusantara*	+	+	+	+	+	−	24	0	+	ATG
*Navicula ramosissima* voucher 10.TA439	+	+	+	+	+	+	23	0/5	+	ATG
*Phaeodactylum tricornutum* strain ICE-H	+	+	+	+	+	−	24	0/4	+	ATG
*Phaeodactylum tricornutum*	+	+	+	+	+	−	23	0/4	+	ATG
*Proschkinia* sp. SZCZR1824	+	+	+	+	+	+	24	0/4	+	ATG
*Surirella* sp.	+	+	+	+	+	−	22	0	+	ATG
Endosymbiont of *Kryptoperidinium foliaceum* (partial)	+	+	+	+	+	−	22	2/1	+	ATA
Endosymbiont of *Durinskia baltica* (partial)	+	+	+	+	+	−	23	0	+	ATG
*Eunotia naegelii* strain UTEX FD354	+	+	+	+	+	−	23	0/1	+	ATG

*The 34 core genes included 32 PCGs (atp6, 8, 9; cob; cox1, 2, 3; nad1-7, 4L, 9, 11; rpl2, 5, 6, 14, 16; rps3, 4, 8, 10, 11, 13, 14, 19; and tatA, tatC) and two rRNAs (rnl and rns).*

### Phylogenetic Analysis of Evolutionary Relationships

**FIGURE 4:**
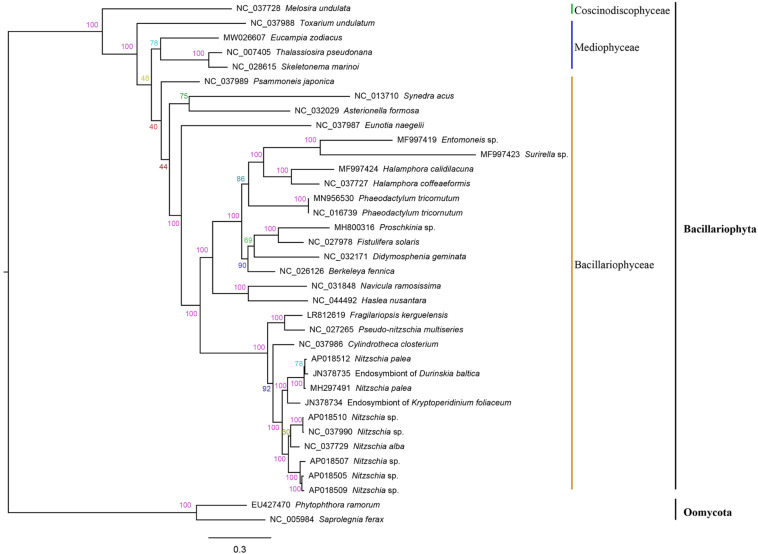
Phylogenetic tree based on maximum likelihood (ML) analysis of amino acid (aa) sequence dataset of 32 mitochondrial PCGs in Bacillariophyta. *Phytophthora ramorum* and *Saprolegnia ferax* were used as out-group taxa. Numbers on the branches represent bootstrap values and Bayesian posterior probabilities, respectively.

The amino acid sequence alignment of 32 concatenated PCGs (5,836 bp combined size) which were shared by mtDNAs of Bacillariophyta and Oomycota was constructed for phylogenetic analysis. Phylogenetic analysis indicated that the 34 species in Bacillariophyta formed three groups, corresponding to three classes of Bacillariophyta, including Bacillariophyceae, Mediophyceae and Coscinodiscophyceae (Figure 4), which was consistent to the current classification in AlgaeBase. *E. zodiacus* belongs to class Mediophyceae that also includes *T. pseudonana*, *S. marinoi*, and *Toxarium undulatum*. *E. zodiacus* formed an independent clade, so did the *T. undulatum*, which was consistent with previous report that *T. pseudonana* was more closely related to *S. marinoi* (An et al., 2017).

**FIGURE 5:**
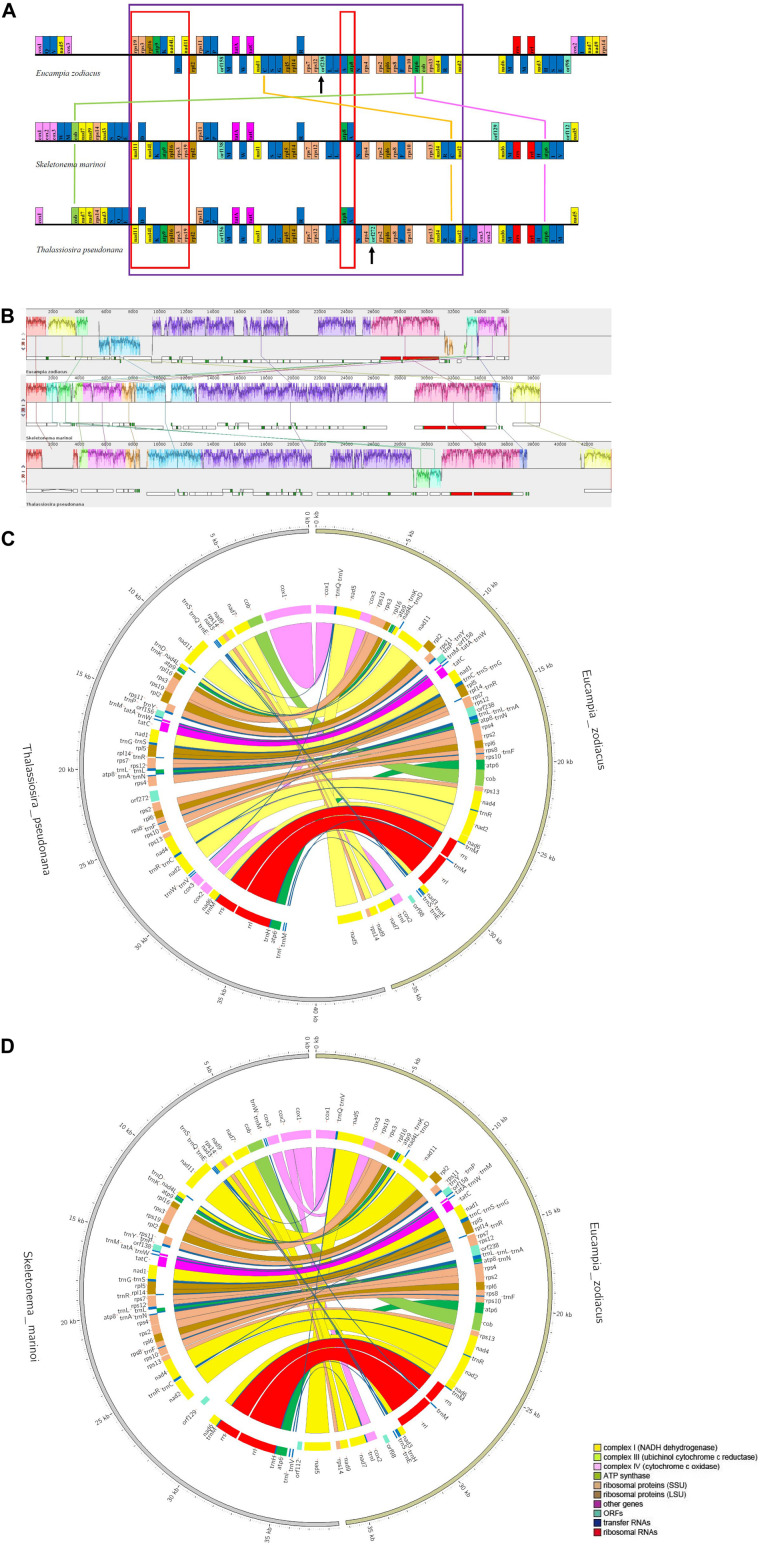
Synteny comparison of *E. zodiacus*, *T. pseudonana* and *S. marinoi* mtDNAs. **(A)** Purple box indicates conserved synteny block of genes, while red box indicates inversion event. Genes with same color share similar function. **(B)** Comparison of *E. zodiacus*, *T. pseudonana* and *S. marinoi* mtDNAs using Mauve. **(C)** CIRCOS plots show synteny comparison between *E. zodiacus* and *T. pseudonana* mtDNAs. Genes with same color share similar function. **(D)** CIRCOS plots show synteny comparison between *E. zodiacus* and *S. marinoi* mtDNAs. Genes with same color share similar function.

Syntenic analysis between *E. zodiacus* and each of *T. pseudonana* and *S. marinoi* revealed a series of translocation and inversion events (Figure 5). High similarity was observed between *T. pseudonana* and *S. marinoi* mtDNAs, with only 5 small translocation events, involving *cox2*, *cox3*, *trnW*, *trnV*, and *trnM*, and several free-standing *orf*s (each being at least 100 codons in size). In contrast, *E. zodiacus* mtDNA exhibited a high level of genome rearrangement when compared to *T. pseudonana* or *S. marinoi*. The three diatom mtDNAs shared a relatively conservative gene block with about 41 genes (from *nad11* to *nad2*), within which gene orders of *T. pseudonana* and *S. marinoi* were almost identical (except for *orf272*). In contrast, *E. zodiacus* had a translocation of *trnC*, a specific *orf238*, and two missing genes (*atp6* and *cob*). What is more, we found that genes in two smaller gene blocks, *nad11-trnD-nad4L-trnK-atp9-rpl16-rps3-rps19* and *atp8-trnA* were inverted in *E. zodiacus* mtDNA.

### Defining a High-Resolution Molecular Marker for Distinguishing *E. zodiacus* Strains

**FIGURE 6:**
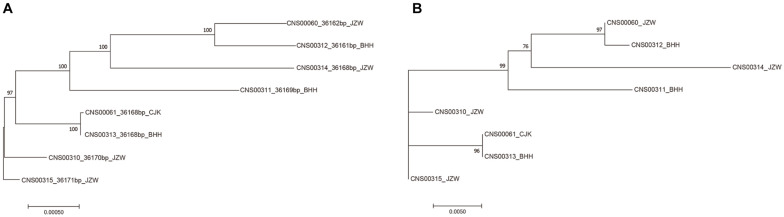
Phylogenetic trees based on maximum likelihood (ML) analysis of eight *E. zodiacus* strains. **(A)** Phylogenetic analysis using the whole mtDNAs of eight *E. zodiacus* strains. **(B)** Phylogenetic analysis using the newly-developed *ezmt1*.

**FIGURE 7:**
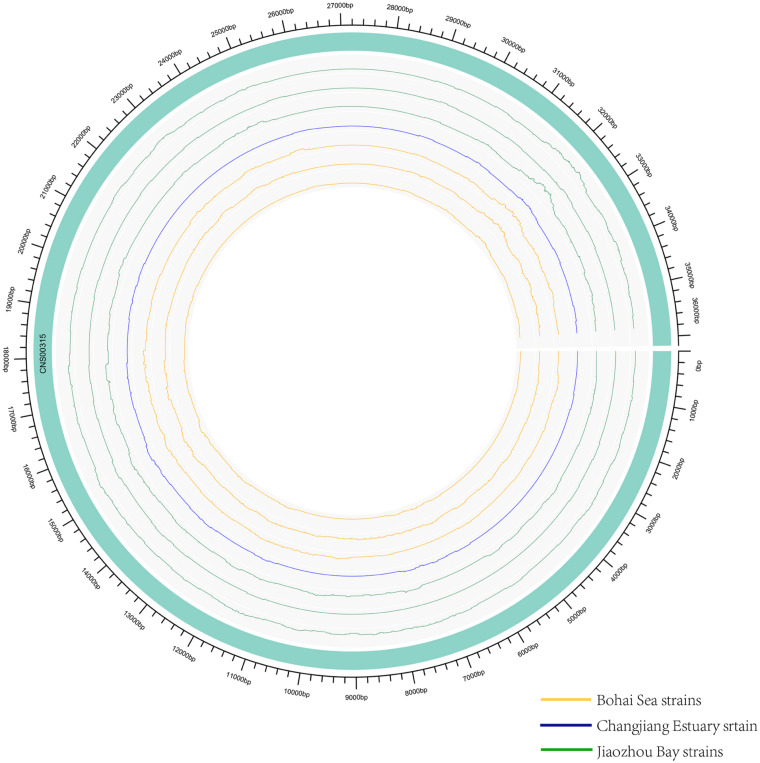
Genomic variations density in *E. zodiacus* strains. The green band represented the reference genome CNS00315. From inside to outside, circles represent three *E. zodiacus* strains isolated from the Bohai Sea (orange), one strain from the Changjiang Estuary (blue), and three strains from the Jiaozhou Bay (green).

While common molecular marker sequences were indistinguishable among these eight *E. zodiacus* strains (Supplementary Figure S1), phylogenetic tree constructed using the whole mtDNAs showed clear between-strain differences (Figure 6A). Comparative analysis of the *E. zodiacus* mtDNAs identified a 400 bp-window with dense variations (Figure 7). We identified 26 SNVs (position: 32,131–32,530 bp in mtDNA of strain CNS00315). This 400 bp region partially overlapped with *orf98* (261 bp). Phylogenetic analysis using this small region of all eight *E. zodiacus* strains based on the sequence alignment suggested that it could be used to effectively distinguish these strains as molecular marker (Figure 6B). The resolution of this molecular marker, which we named *E. zodiacus* mitochondrial 1 and abbreviated as *ezmt1*, was high.

### Specificity Evaluation of *ezmt1*

The specificity of a molecular marker is high if it can be used only for distinguishing a small set of closely related species. In contrast, the specificity of a molecular marker is low if it can be used for distinguishing a large set of broadly related species. In this study, we would like to identify a molecular marker with high specificity that specifically recognizes intra-species variations in the species *E. zodiacus*. To test the specificity of newly developed molecular marker *ezmt1*, we first carried out BLAST searches against the NCBI nt database, which showed low similarity and low coverage to sequences of other species. Second, we carried out PCR amplification assays on DNAs extracted from 13 representative eukaryotic algae species, including seven species in Bacillariophyta including *S. marinoi*, *Thalassiosira weissflogii*, *Chaetoceros curvisetus*, *Pseudo-nitzschia pungens*, *Planktoniella sol*, *Psammodictyon constrictum*, and *Rhizosolenia* sp., three species in Dinoflagellata including *Alexandrium tamarense*, *Karenia mikimotoi*, and *Prorocentrum donghaiense*, three species in Ochrophyta including *Aureococcus anophagefferens*, *Chattonella marina*, and *P. globosa*. Results of all 13 PCR reactions showed that *ezmt1* sequences could only be amplified in *E. zodiacus* (Supplementary Figure S2), further confirming the high specificity of *ezmt1*.

## Discussion

The *E. zodiacus* is a common HAB species that has been identified in many ocean regions including the Tokyo Bay (Nishikawa et al., 2011), Harima-Nada (Nishikawa et al., 2007), and Ariake sea (Matsubara, 2012) in Japan, Bay of Fundy (Martin et al., 2008) in Canada, Jiaozhou Bay, Haizhou Bay, Xiangshan Harbour and many other sea areas in China (Huo et al., 2001; Zhang et al., 2002; Liang, 2012). Indeed, it is the only HAB species that has been identified in all recorded expeditions in the Jiaozhou Bay (Liu and Chen, 2021). *E. zodiacus* HABs have been found to have caused negative impacts on bleaching of aquacultured nori, fisheries damage and economic losses (Martin et al., 2008; Nishikawa et al., 2011). The differential special and temporal features of *E. zodiacus* HABs reported in Japan (Nishikawa et al., 2007) and China (Huo et al., 2001; Zhang et al., 2002; Liang, 2012) suggest that it has important genetic diversity. Nevertheless, the genomic information of *E. zodiacus* is limited and the genetic diversity of *E. zodiacus* has not been studied.

In this project, we constructed the mtDNA of *E. zodiacus* for the first time, which was also the first mitochondria genome for all species in the order Hemiaulales. The mtDNA of *E. zodiacus* was 36,162 bp in size, which is shorter than most diatom mtDNAs that are generally compact with few repeats and a small number of introns (Secq and Green, 2011). The small size of mtDNA of *E. zodiacus* is due to its small intergenic regions, the low repeat content and the absence of introns. First, the variations in mtDNA sizes could be due to variations of intergenic regions (Pogoda et al., 2019), and the average intergenic regions for *T. pseudonana* and *Phaeodactylum tricornutum* (Secq and Green, 2011) are 157 and 841 bp, respectively. The average length of intergenic regions of *E. zodiacus* mtDNA was only 39 bp. Second, repeats in diatom mtDNAs are either small or concentrated in only a small number of sites, without interrupting the genes in the mtDNAs or gene densities of the mtDNAs. For example, only a single 35 kb-long repeat was found in the mtDNA of *P. tricornutum* (Secq and Green, 2011). No such repeats were found in the mtDNA of *E. zodiacus*. Third, the introns in the diatom mtDNAs are generally found in a few genes including *cox1* (Guillory et al., 2018), *rnl*, *rns*, *cob*, *cox2*, *cox3*, and *nad7* (Pogoda et al., 2019). No introns were found in *E. zodiacus* mtDNA.

There is very little difference in gene content of diatom mtDNAs, except for *orf*s, some of which are found within introns (Pogoda et al., 2019). The only gene that was not found in the *E. zodiacus* mtDNA was *rrn5*, which is found only in a few diatom species (Secq and Green, 2011; Valach et al., 2014). The *rrn5* may exist in the common ancestor of organelle genomes but have lost afterward (Valach et al., 2014). A full *nad11* gene was found in the *E. zodiacus* mtDNA. This gene is present in many diatom mtDNAs including the mtDNAs of *T. undulatum* (Guillory et al., 2018) and *Asterionella formosa* (Villain et al., 2017), while it is split into two parts in the mtDNAs of many species in Bacillariophyceae including *Cylindrotheca closterium* (Guillory et al., 2018) and *Nitzschia palea* (Crowell et al., 2019). Three ribosomal protein coding genes *rps2*, *rps7*, and *rps12* are lost in some diatom mtDNAs (Pogoda et al., 2019). However, all of these three genes are found in the *E. zodiacus* mtDNA.

The advantage of compact *E. zodiacus* mtDNA is not known (Secq and Green, 2011; Liu et al., 2014). However, as intergenic regions may facilitate intragenomic recombination, as observed in mtDNAs of mosses (Liu et al., 2014), the small intergenic regions in *E. zodiacus* mtDNA may be associated with low intragenomic recombination activities, which may be critical for maintaining the stability of the mtDNA. Furthermore, the organization of genes is important to the transcription of polycistronic operons (Liu et al., 2014), thus the small genome size of *E. zodiacus* mtDNA may be important in insuring proper transcription of genes in the mtDNA.

While the number of genes in diatom mtDNAs show high similarity, their syntenic relationships vary greatly in a lineage-specific manner. Numerous genome rearrangement events were observed between *E. zodiacus* and mtDNAs of other diatom species, which may be explained by the large evolutionary distances between *E. zodiacus* and other diatom species. However, the mtDNA of *E. sodiacus* shared relatively high syntenic similarity with mtDNAs of representative diatoms including *T. pseudonana* and *S. marinoi* (Figures 5A,B) of another order in class Mediophyceae, supporting the current taxonomic status of *E. zodiacus*, which is also supported by phylogenetic analysis based on core genes. mtDNAs of more closely related species are needed to understand the origin and the evolutionary relationship of such genome rearrangements.

An ideal molecular marker usually requires many criteria. First, low intra-genome variation among multiple copies of a molecular marker is critical for ensuring enough representativeness and reduce ambiguity (Xiao-Kun et al., 2019). Second, a molecular marker should have conserved flanking sequences to facilitate the design of universal primers and obtain an appropriate sequence length in a single amplification (Guo et al., 2015). For example, the short variable region V4 region of the 18S rDNA sequence, which is frequently used for metabarcoding analysis of microbial eukaryotes (Decelle et al., 2014; Liu et al., 2020). Third, a molecular marker should have appropriate specificity, dependent on its applications (Fechner et al., 2010). To be specific, when surveying large number of species in large areas, low specificity is preferred. When focusing on specific species, like in this project, for tracking *E. zodiacus* strains, high specificity is more desirable.

For this project, we isolated and characterized eight *E. zodiacus* strains from three different sea areas in China, spanning about eight latitudes (30.3625°N–38.3658°N) and covering three seasons (summer, autumn, and winter). Despite such large geographical span and seasonal differences, phylogenetic analysis based on common molecular markers could not distinguish these strains, suggesting that they shared high genetic similarity. We found clear distinction among different *E. zodiacus* strains based on whole mtDNAs, suggesting unambiguous genetic differences among different *E. zodiacus* strains. Through sequence alignment and comparative analysis, we identified a molecular marker *ezmt1* that could adequately distinguish different *E. zodiacus* strains. Common molecular markers of *E. zodiacus* may fit part of the criteria listed above, while *ezmt1* satisfies all criteria. The *ezmt1* could be an effective molecular marker for studying *E. zodiacus* all over the world. On the one hand, we can distinguish and track different strains of *E. zodiacus*, especially during blooms, to evaluate strain-specific differential contribution to blooms. For example, *E. zodiacus* blooms occurred in Japan in winter (Nishikawa and Yamaguchi, 2006; Nishikawa et al., 2009) revealed different features with that in China usually occurred in summer (Huo et al., 2001; Zhang et al., 2002; Liang, 2012), which suggested that different *E. zodiacus* strains involved. The newly designed molecular marker *ezmt1* may help study the genetic evolutionary relationship between them. On the other hand, by further collecting large number of samples, we can study the geographical distribution pattern of *E. zodiacus* strains.

## Conclusion

*E. zodiacus* is the first species having its complete mitogenome sequenced in the order Hemiaulales. The availability of the *E. zodiacus* mtDNA will facilitate evolutionary study of mtDNAs in Bacillariophyta, especially in the order Hemiaulales. Through comparative analysis of mtDNAs among different *E. zodiacus* strains, we identified a molecular marker *ezmt1* that can effectively distinguish different *E. zodiacus* strains. The *ezmt1* holds great potential in research on genetic diversity in *E. zodiacus*, and, more importantly, on tracking causative strain in *E. zodiacus* HABs.

## Data Availability Statement

The original contributions presented in the study are publicly available. This data can be found here: https://www.ncbi.nlm.nih.gov/sra/PRJNA682714 and https://www.ncbi.nlm.nih.gov/nuccore/MW026607.

## Author Contributions

MZ and NC conceived and designed the experiments. MZ and ZC collected the samples. MZ performed the experiments and wrote the manuscript. MZ, FL, and NC analyzed the data. MZ, ZC, FL, and NC read and approved the manuscript. All authors contributed to the article and approved the submitted version.

## Conflict of Interest

The authors declare that the research was conducted in the absence of any commercial or financial relationships that could be construed as a potential conflict of interest.
