# Supplementary material for: Definition of a High-Resolution Molecular Marker for Tracking the Genetic Diversity of the Harmful Algal Species Eucampia zodiacus Through Comparative Analysis of Mitochondrial Genomes
Source: Front Microbiol. 2021 Mar 24;12:631144. doi: 10.3389/fmicb.2021.631144 (PMC8024477; doi:10.3389/fmicb.2021.631144)
Supplement: Supplementary file 1 [file Data_Sheet_1.PDF]

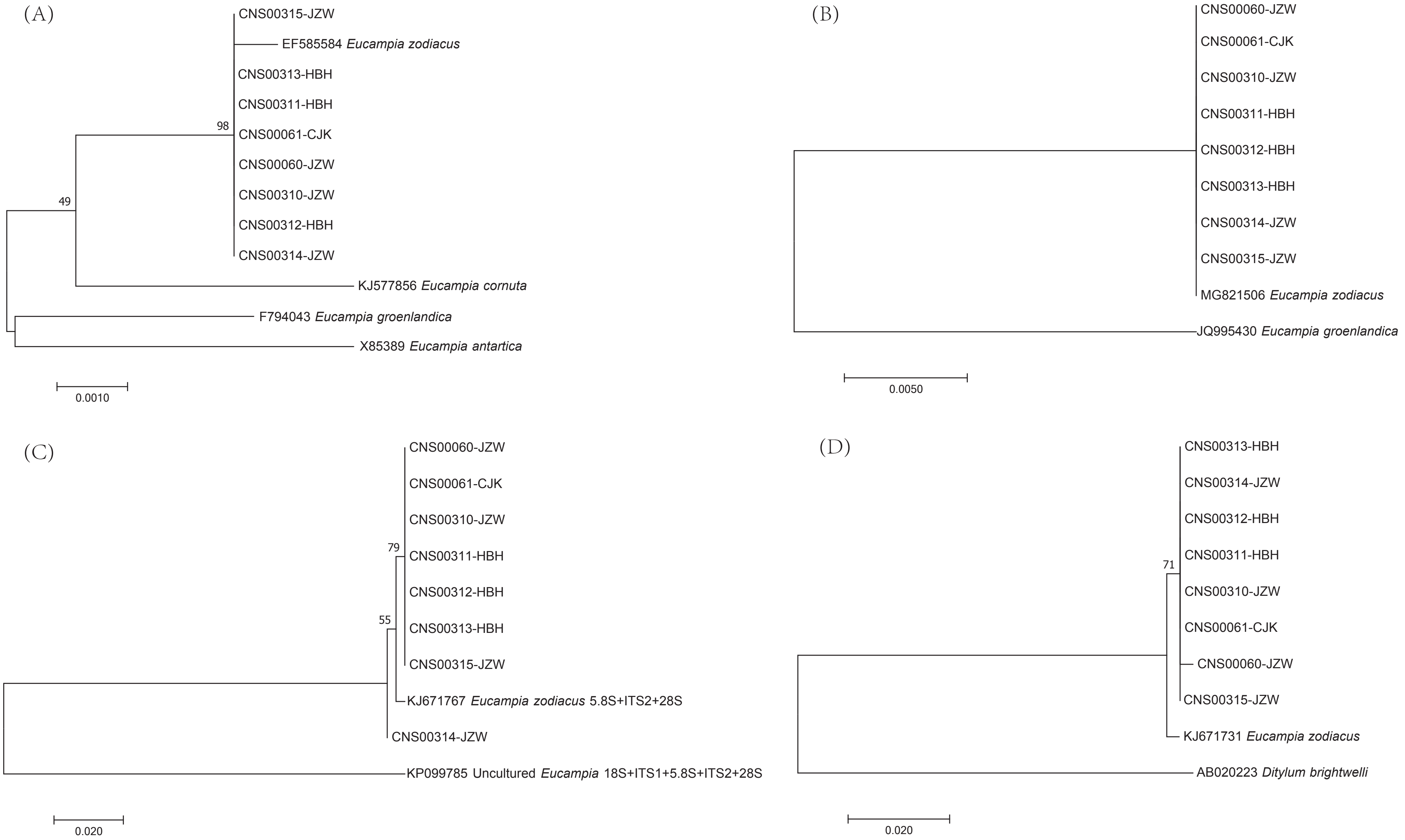

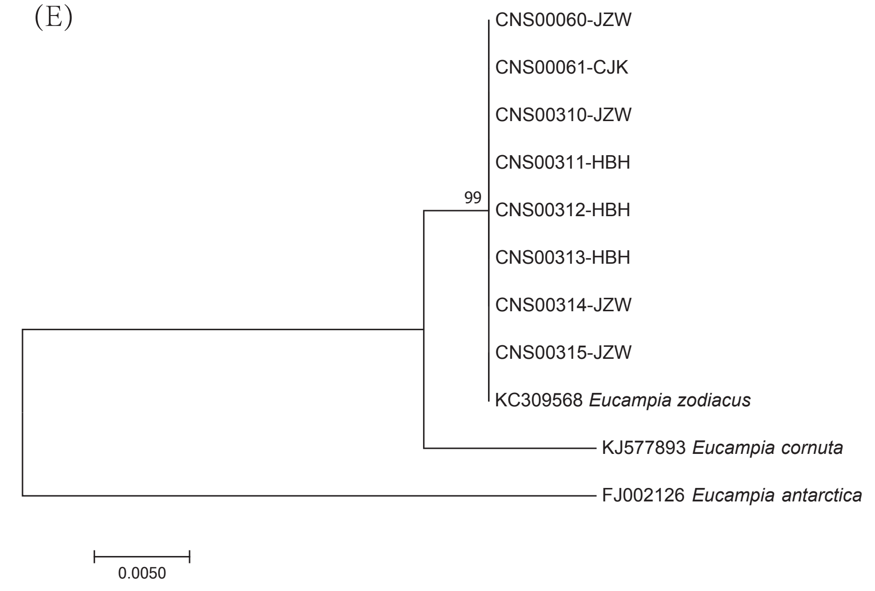

Figure S1. Phylogenetic trees based on common molecular marker sequences.
(A) Phylogenetic tree based on 18S rDNA.
(B) Phylogenetic tree based on 28S rDNA D1-D2.
(C) Phylogenetic tree based on ITS.
(D) Phylogenetic tree based on *COI*.
(E) Phylogenetic tree based on *rbcL*.
