# Supplementary material for: Definition of a High-Resolution Molecular Marker for Tracking the Genetic Diversity of the Harmful Algal Species Eucampia zodiacus Through Comparative Analysis of Mitochondrial Genomes
Source: Front Microbiol. 2021 Mar 24;12:631144. doi: 10.3389/fmicb.2021.631144 (PMC8024477; doi:10.3389/fmicb.2021.631144)
Supplement: Supplementary file 2 [file Data_Sheet_2.PDF]

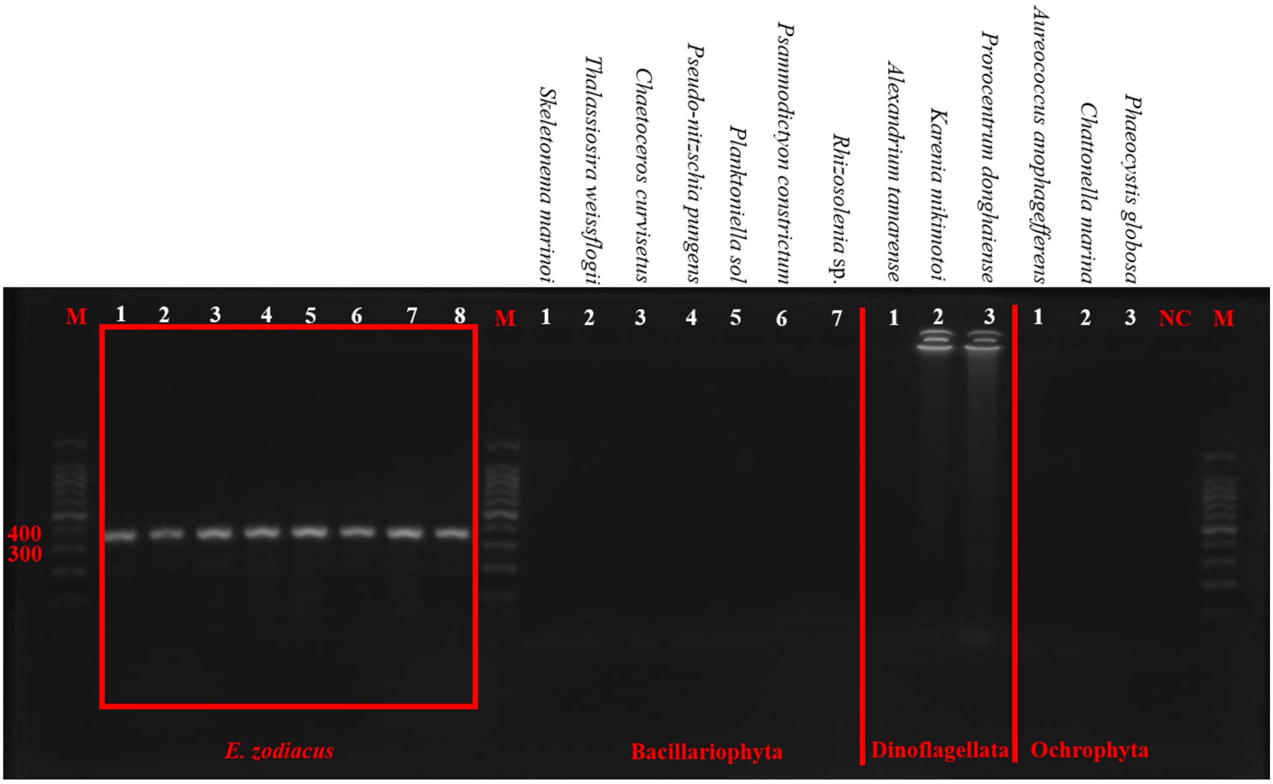

Figure S2. PCR amplification assays on 13 representative eukaryotic algae species.

The assay included seven species in Bacillariophyta including *Skeletonema marinoi*, *Thalassiosira weissflogii*, *Chaetoceros curvisetus*, *Pseudo-nitzschia pungens*, *Planktoniella sol*, *Psammodictyon constrictum* and *Rhizosolenia* sp., three species in Dinoflagellata including *Alexandrium tamarense*, *Karenia mikimotoi* and *Prorocentrum donghaiense*, three species in Ochrophyta including *Aureococcus anophagefferens*, *Chattonella marina* and *Phaeocystis globosa*. *Ezmt1* sequences could only be amplified in *E. zodiacus*.
